# RHGF-2 Is an Essential Rho-1 Specific RhoGEF that binds to the Multi-PDZ Domain Scaffold Protein MPZ-1 in *Caenorhabditis elegans*

**DOI:** 10.1371/journal.pone.0031499

**Published:** 2012-02-20

**Authors:** Li Lin, Thuy Tran, Shuang Hu, Todd Cramer, Richard Komuniecki, Robert M. Steven

**Affiliations:** Department of Biological Sciences, University of Toledo, Toledo, Ohio, United States of America; Alexander Flemming Biomedical Sciences Research Center, Greece

## Abstract

RhoGEF proteins activate the Rho family of small GTPases and thus play a key role in regulating fundamental cellular processes such as cell morphology and polarity, cell cycle progression and gene transcription. We identified a *Caenorhabditis elegans* RhoGEF protein, RHGF-2, as a binding partner of the *C. elegans* multi-PDZ domain scaffold protein MPZ-1 (MUPP1 in mammals). RHGF-2 exhibits significant identity to the mammalian RhoGEFs PLEKHG5/Tech/Syx and contains a class I C-terminal PDZ binding motif (SDV) that interacts most strongly to MPZ-1 PDZ domain eight. RHGF-2 RhoGEF activity is specific to the *C. elegans* RhoA homolog RHO-1 as determined by direct binding, GDP/GTP exchange and serum response element-driven reporter activity. *rhgf-2* is an essential gene since *rhgf-2* deletion mutants do not elongate during embryogenesis and hatch as short immobile animals that arrest development. Interestingly, the expression of a functional *rhgf-2::gfp* transgene appears to be exclusively neuronal and *rhgf-2* overexpression results in loopy movement with exaggerated body bends. Transient expression of RHGF-2 in N1E-115 neuroblastoma cells prevents neurite outgrowth similar to constitutive RhoA activation in these cells. Together, these observations indicate neuronally expressed RHGF-2 is an essential RHO-1 specific RhoGEF that binds most strongly to MPZ-1 PDZ domain eight and is required for wild-type *C. elegans* morphology and growth.

## Introduction

Members of the Rho family of small GTPases, including RhoA, Rac and Cdc42, play key roles in regulating neuron morphology and function, from axon extension to neurotransmission [1]–[3]. The importance of Rho family GTPase signaling in the nervous system is highlighted by mutations in these pathways, which result in significant cognitive and behavioral defects in humans [4]. Rho family GTPases are primarily regulated by guanine nucleotide exchange factors (RhoGEFs) and GTPase activating proteins (RhoGAPs), which positively and negatively influence Rho GTPase activation, respectively [5]. The number of Rho family GTPase regulators greatly exceeds the number of Rho family GTPases and these modulators of GTPase signaling tend to have tissue specific expression patterns indicating their importance in establishing the correct spatial and temporal activation of the widely expressed Rho family GTPases.

RhoGEFs activate Rho family GTPases by facilitating GDP/GTP exchange and a number of nervous system specific RhoGEFs have been identified including Collybistin, the *Drosophila* Tiam1 homolog Still Life (SIF) and specific isoforms of intersectin and the Trio/Kalirin/UNC-73 family of proteins [6]–[15]. More recently, a new family of RhoA specific RhoGEFs with C-terminal PDZ (Postsynaptic Density 95, Disks Large, Zona Occludens-1)-binding motifs was identified that includes human PLEKHG5 (Pleckstrin Homology Domain–Containing, Family G, Member 5), mouse/zebrafish Syx (Synectin-Binding Guanine Exchange Factor) and rat Tech (Transcript Enriched in Cortex and Hippocampus). PLEKHG5, also known as GEF720, is expressed specifically in the brain and PLEKHG5 activity blocks the ability of NGF to induce neurite outgrowth in PC12 cells, consistent with RhoA activation [16], [17]. Mutations in the *PLEKHG5* gene are associated with a form of degenerative lower motor neuron disease, revealing its importance in the nervous system [18]. Tech expression is enriched in the cortex and hippocampus of the rat brain and Tech activity decreases the number of dendritic processes in cultured cortical neurons [19]. Genetic analysis of mouse/zebrafish Syx, on the other hand, indicates Syx is required early in development for anterior/posterior extension of the zebrafish embryo as well as endothelial cell migration and tube formation during angiogenesis [20]–[24]. Interestingly, a low abundance Syx splice variant, Syx2, which lacks a PDZ-binding motif, is highly expressed in glioblastoma multiforme, a common and highly malignant primary brain tumor [22].

PLEKHG5/Tech/Syx function is regulated by several proteins. For the spatial control of Rho GTPase activity in migrating endothelial cells, Syx binds to the single PDZ domain-containing protein, synectin, as well as the scaffold protein MUPP1 (Multi PDZ Domain Protein 1) and its paralog Patj (Protein Associated with Tight Junctions) via the PDZ-binding motif at the Syx C-terminus [20], [22], [25]. Tech also binds MUPP1 in cortical neurons and is proposed to localize Rho activity at synapses [26]. Negative regulation of Syx activity occurs during gastrulation via Rnd3, a member of the unique Rnd subfamily of Rho GTPases, which binds to the Syx RBD-like (Ras Binding Domain) region [24]. These observations highlight the importance of PLEKHG5/Tech/Syx localization in development, both early, during gastrulation, and later for neuronal development and angiogenesis.

In this study we identified a *Caenorhabditis elegans* PLEKHG5/Tech/Syx homolog, RHGF-2 (RhoGEF Protein 2), as a binding partner for the *C. elegans* multi-PDZ domain scaffold protein MPZ-1 (Multiple PDZ Domain Protein 1). MPZ-1 is closely related to the mammalian PDZ domain scaffold protein MUPP1 [27]. RHGF-2 contains a class I C-terminal PDZ-binding motif (SDV) and binds strongly to MPZ-1 PDZ domain eight in a PDZ binding motif-dependent manner. The expression of functional RHGF-2::GFP is confined to a subset of *C. elegans* neurons and RHGF-2 overexpression leads to altered locomotion with a higher amplitude waveform than wild type. RHGF-2 GEF activity is specific to RHO-1, the *C. elegans* RhoA homolog and transient expression of RHGF-2 prevents neurite outgrowth in N1E-115 cells similar to the activity of activated RhoA in these cells [28], [29]. Importantly, RHGF-2 is essential for development as *rhgf-2* deletion mutants arrest development without elongating and hatch as short immobile animals. Together, these observations suggest that the many experimental advantages of the *C. elegans* model organism may be useful in understanding the factors modulating mammalian PLEKHG5/Tech/Syx signaling.

## Results

### Identification of RHGF-2 as an MPZ-1 binding partner

**Figure 1:**
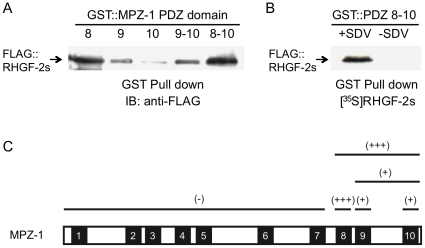
RHGF-2 binds most strongly to MPZ-1 PDZ domain eight in a PDZ-binding motif dependent manner. *A*. Purified GST::MPZ-1 PDZ domains were incubated with the lysates of HEK293T cells transfected with FLAG::RHGF-2s. GST fusion proteins were pulled down with glutathione-sepharose beads and bound proteins were separated by SDS-PAGE and analyzed by immunoblotting with anti-FLAG antibody. *B*. [^35^S]-labeled FLAG::RHGF-2s, with or without the C-terminal PDZ-binding motif (SDV), was incubated with GST::MPZ-1(PDZ domains 8–10). Glutathione-sepharose beads were used to pull down the GST fusion protein and bound proteins were resolved by SDS-PAGE and visualized by autoradiography. *C*. A summary of the interactions between RHGF-2s and fragments of MPZ-1 as examined by yeast two-hybrid and protein pull-down experiments. Full length MPZ-1 with PDZ domains represented by numbered boxes is at the bottom of the figure. MPZ-1 fragments are represented by lines above the full-length protein and the affinity of RHGF-2s to each fragment is categorized as either strong (+++), weak (+) or no affinity (−).

To identify potential binding partners for the *C. elegans* multi-PDZ domain scaffold protein MPZ-1, distinct PDZ domain-containing fragments of MPZ-1 were used as bait in yeast two-hybrid screens of a mixed-stage *C. elegans* hermaphrodite cDNA library (Materials and Methods). Using MPZ-1 PDZ domains 8–10 we identified T08H4.1, which we named RHGF-2 (Rho Guanine Nucleotide Exchange Factor-2; see below), as a potential MPZ-1 interacting protein. This interaction was confirmed, and the RHGF-2 binding site on MPZ-1 was mapped, using recombinant proteins. GST::MPZ-1 PDZ domain fusion proteins bound to glutathione-sepharose beads were incubated with lysates from HEK293T cells expressing FLAG::RHGF-2. Bound proteins were eluted from the beads, separated by SDS-PAGE and immunoblotted with anti-FLAG antisera (Fig. 1). RHGF-2 bound strongly to MPZ-1 PDZ domain 8 and to a lesser extent to PDZ domains 9 and 10, but not at all to PDZ domains 1–7 (Fig. 1A). Importantly, the interaction between MPZ-1 PDZ domains 8–10 and RHGF-2 required the RHGF-2 C-terminal class I PDZ-binding motif (SDV) [30]. Radiolabeled RHGF-2 and C-terminally truncated RHGF-2 without the PDZ-binding motif were produced by *in vitro* transcription/translation reactions and incubated with a GST::MPZ-1 fragment containing PDZ domains 8–10. Only RHGF-2 containing the PDZ-binding motif bound to the MPZ-1 PDZ domains in a GST pull down experiment (Fig. 1B).

### RHGF-2 is a member of the PLEKHG5/Tech/Syx family of RhoGEFs

**Figure 2:**
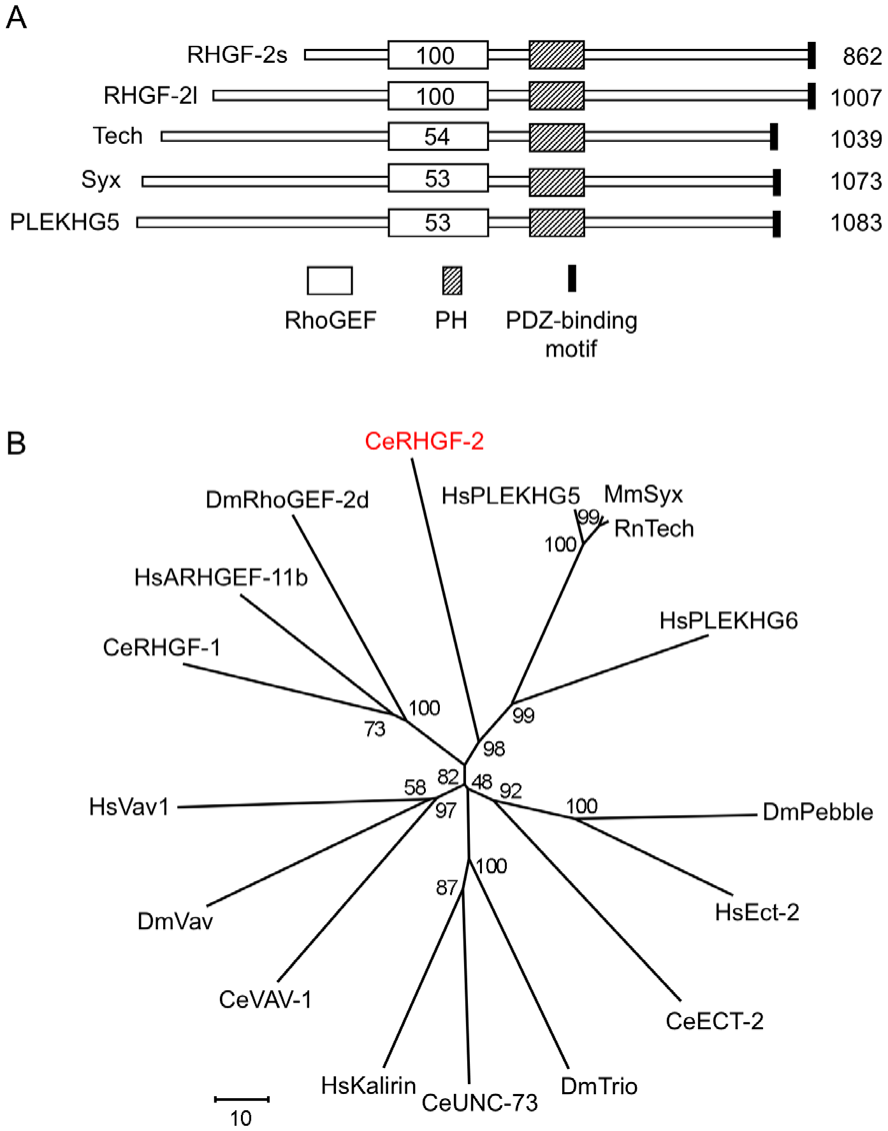
RHGF-2 is similar to the PLEKHG5/Syx/Tech RhoGEF family of proteins. *A*. Domain structures of RHGF-2 and its putative homologs rat Tech, mouse Syx and human PLEKHG5. The percent similarity between the sequences of the RhoGEF domains of RHGF-2 and the homologs are indicated. The percent identity between the domains is ∼31%. *B*. Phylogenetic tree comparing the RhoGEF domains from selected proteins. The RhoGEF domains were defined by SMART (http://smart.embl-heidelberg.de/) [92]. Sequence analysis (ClustalW), bootstrapping (500 replicates) and tree compilation (neighbor-joining method) was performed using MEGA 5 software [93]. *C. elegans* protein sequences were obtained from WormBase.org, all other sequences are from NCBI. Ce *Caenorhabditis elegans* (ECT-2: NP_496318.1, RHGF-1: NP_509791, RHGF-2: NP_494723.1, UNC-73 (C-terminal RhoGEF): AAC71110.1, VAV-1: NP_001041223.1), Dm *Drosophila melanogaster* (Pebble: NP_729306.1, RhoGEF-2d: NP_477317.1, Trio (C-terminal RhoGEF): NP_728561.1, Vav: NP_573372.1), Hs *Homo sapiens* (ARHGEF-11b: NP_937879.1, Ect-2: NP_060568.3, PLEKHG5: NP_941374.2, PLEKHG6: NP_001138329.1, Kalirin (C-terminal RhoGEF): NP_008995.2, Vav1: AAB34377.1), Mm *Mus musculus* (Syx: NP_001004156) and Rn *Rattus norvegicus* (Tech: NP_958429).

**Figure 3:**
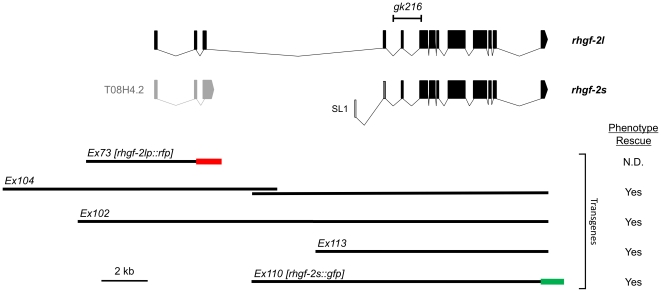
The predicted *rhgf-2* transcripts along with the *rhgf-2* DNA constructs and fragments used in this study. cDNA regions are represented by boxes (exons) and genomic DNA is represented by straight black lines with the corresponding extrachromosomal array name indicated above. The genomic region deleted in *rhgf-2(gk216)* animals is indicated at the top of the diagram. The red and green lines represent tagRFP and GFP sequences, respectively, and each is followed by the *let-858* 3′ UTR (not indicated). cDNA analysis revealed that the WormBase predicted gene T08H4.2 (gray) is actually the 5′ end of *rhgf-2* (Materials and Methods). The predicted full-length *rhgf-2* transcript is indicated as *rhgf-2l*. A shorter, but still functional transcript, with a trans-spliced SL1 leader sequence, is indicated as *rhgf-2s* (Materials and Methods). The diagram is drawn to scale. The ability of a particular array to rescue the *rhgf-2(gk216)* developmental arrest and Dpy phenotypes is indicated in the column on the right. More detailed rescue information is included in Table 1.

The predicted *rhgf-2* open reading frame encodes a protein containing tandem RhoGEF and pleckstrin homology (PH) domains, which are conserved among the GDP/GTP exchange factors for Rho family GTPases (Fig. 2A; wormbase.org) [5], [31]. Alignment of the RhoGEF domains from a number of RhoGEF proteins yielded the phylogenetic tree in Figure 2B, which indicated RHGF-2 is most closely related to the rat Tech, mouse Syx and human PLEKHG5 RhoGEF family. PLEKHG6 (MyoGEF), which functions in cytokinesis and cell migration exhibits approximately 45% identity to PLEKHG5/Syx/Tech family members and 30% identity to the RHGF-2 RhoGEF domain, but it does not appear to be expressed in the nervous system like the PLEKHG5/Syx/Tech proteins [32]–[34]. Since the RHGF-2 protein predicted by WormBase (RHGF-2s in Fig. 2A) had a shorter N-terminus in comparison to the mammalian homologs, we carefully examined cDNAs corresponding to the 5′ end of the *rhgf-2* gene (Materials and Methods). This analysis resulted in the identification of additional 5′ *rhgf-2* coding sequence and a correspondingly larger predicted RHGF-2 protein (RHGF-2l, for long isoform; Figs. 2 and 3). Although the additional RHGF-2 N-terminal amino acid sequence did not show significant identity to the N-terminal regions of the mammalian RHGF-2 homologs, it brought RHGF-2 to a comparable length (Fig. 2).

### RHGF-2 has RhoGEF activity specific to RHO-1

**Figure 4:**
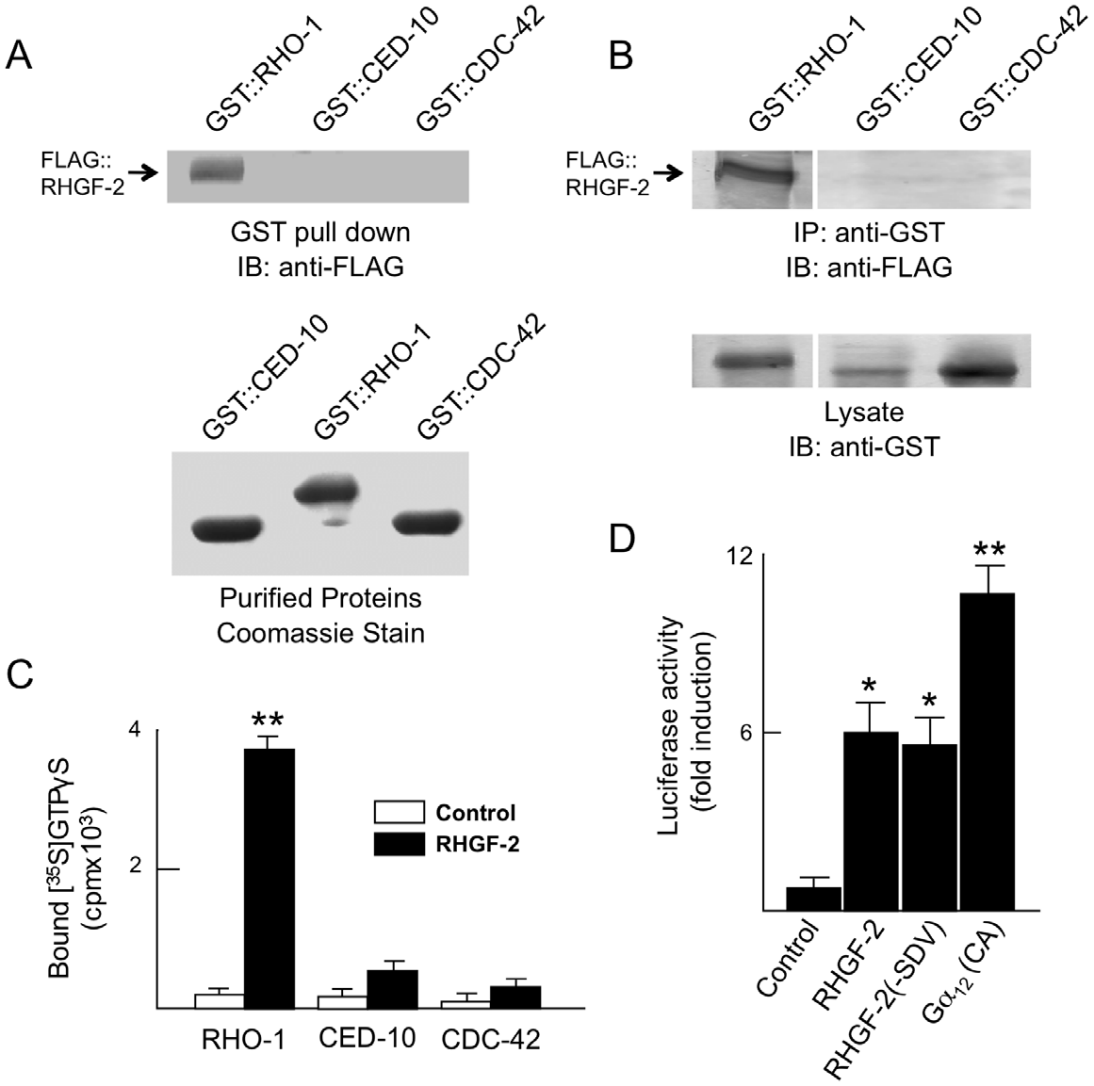
RHGF-2 binds to and activates *C. elegans* RHO-1. *A*. RHO-1, CDC42 and CED-10 GST fusion proteins were purified on glutathione sepharose (lower panel). The purified fusion proteins were incubated with lysates from cells expressing FLAG::RHGF-2s and collected on glutathione sepharose beads. Bound proteins were separated by SDS-PAGE and analyzed by immunoblotting with anti-FLAG antibody (upper panel). *B*. *Flag::rhgf-2s* was transiently cotransfected with *rho-1*, *ced-10* or *cdc-42* GST fusions. Cell lysates prepared 48 hours after transfection were immunoprecipitated with anti-GST antibody, and immunoprecipitates were analyzed by immunoblotting with anti-FLAG antibody (upper panel). Cell lysates were immunoblotted with anti-GST to confirm the expression of the appropriate fusion protein (lower panel). *C*. RHGF-2s activity is specific to RHO-1. Purified RHO-1, CDC-42 or CED-10 GST fusions preloaded with unlabelled GDP were incubated in the presence (black bars) or absence (white bars) of FLAG-tagged RHGF-2s immunoprecipitated from transiently transfected HEK293T cells. The GEF assay was performed in the presence of 0.1 nM [^35^S]GTPγS, which was assayed for GTPase binding by filtration on nitrocellulose. *D*. RHGF-2s stimulates SRE activation in HEK293T cells. HEK293T cells were co-transfected with *rhgf-2s* together with pMCV-β-galactosidase and SRE-luciferase reporter plasmids. Total amounts of DNA were kept constant with empty vector. After 24 hours cells were transferred to DMEM without serum and after an additional 24 hours they were lysed and assayed. Error bars indicate SEM. **p<0.001 and *p<0.01 in comparison to control reactions using Student's *t*-test.

To investigate the biochemical activity and specificity of RHGF-2 we first examined RHGF-2 binding to *C. elegans* homologs of the prototypical members of the three major Rho GTPase subgroups, RhoA (RHO-1), Rac (CED-10) and Cdc42 (CDC-42) [35]. FLAG::RHGF-2 was expressed in HEK293T cells and binding to Rho GTPases was assessed by GST pull down experiments using purified GST::RHO-1, GST::CED-10 or GST::CDC-42 (Fig. 4A). FLAG::RHGF-2 bound preferentially to GST::RHO-1 in comparison to binding with GST::CED-10 or GST::CDC-42. A similar result was obtained when FLAG::RHGF-2 binding to Rho-family GTPases was assessed by co-immunoprecipitation of the proteins co-expressed in HEK293T cells (Fig. 4B).

Next, RHGF-2 RhoGEF activity was assayed directly with the same *C. elegans* Rho family GTPases. FLAG::RHGF-2 expressed in HEK293T cells was immunoprecipitated from cell lysates with anti-FLAG antibody and used for *in vitro* GEF assays with purified RHO-1, CDC-42 and CED-10 GST fusion proteins. As shown in Figure 4C, FLAG::RHGF-2 strongly stimulated the exchange of GDP for GTPγS^35^ on GST::RHO-1, but not on GST::CDC-42 or GST::CED-10.

Finally, since activated RhoA effectively stimulates transcription through serum response factor (SRF) and the serum response element (SRE) [36], we examined the ability of transiently expressed *rhgf-2* to activate RhoA by monitoring SRE activation. *rhgf-2* and a luciferase reporter under the control of an SRE were co-expressed in HEK293T cells and luciferase activity was measured. Since an activated form of the heterotrimeric G protein Gα_12_ also stimulates SRE-mediated transcription through RhoA and SRF [36], we used activated Gα_12_ as a positive control. As shown in Figure 4D, constitutively active Gα_12_ dramatically induced luciferase activity and, under identical conditions, RHGF-2 also elevated luciferase activity about 6-fold. Deletion of the RHGF-2 PDZ-binding motif had no effect on its ability to stimulate luciferase activity (Fig. 4D).

### *rhgf-2* mutants arrest development and do not elongate

**Figure 5:**
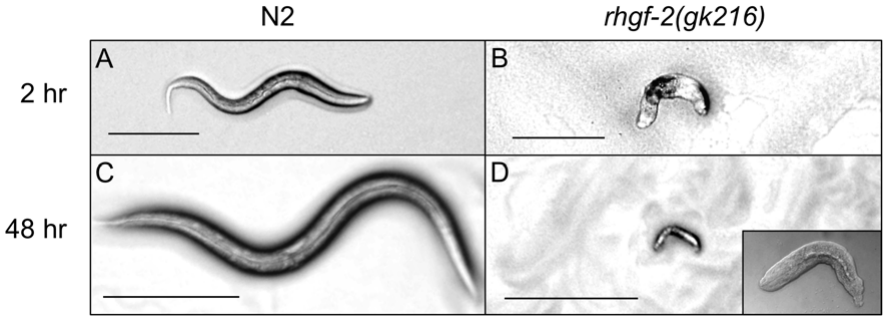
*rhgf-2(gk216)* deletion mutants arrest development and hatch as short immobile animals. Representative wild-type N2 (A) and *rhgf-2(gk216)* (B) animals approximately two hours after hatching. Scale bar is 100 µm. N2 (C) and *rhgf-2(gk216)* (D) animals approximately 48 hours after hatching. Scale bar is 200 µm. Another *rhgf-2(gk216)* mutant is visible in more detail in a differential interference contrast image (inset).

The recessive *gk216* mutation, generated by the *C. elegans* Knockout Consortium, is a 1381 bp deletion within the *rhgf-2* gene that removes 141 bp of early *rhg1f-2* coding sequence (Fig. 3). This deletion is expected to severely disrupt the production of RHGF-2 protein(s) by placing most of the coding sequence including the RhoGEF domain, out of frame. Homozygous *gk216* mutants arrest development after hatching as immobile and morphologically abnormal animals with a severe Dpy phenotype (dumpy; shorter than wildtype) (Fig. 5). Examination of the progeny phenotypes from a balanced *rhgf-2(gk216)/mIn1* strain revealed 25.6% arrested L1 larvae, 73.4% wild-type and 1.0% unhatched eggs (n = 312).

**Figure 6:**
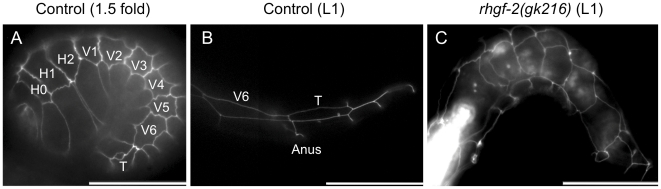
Hypodermal (epidermal) cells in *rhgf-2(gk216)* mutants do not elongate. The apical cell junction marker AJM-1::GFP was used to examine hypodermal cell morphology in control and *rhgf-2(gk216)* mutant backgrounds at the indicated stages of development. Hypodermal seam cells in control animals are square or rounded in shape at the 1.5 fold stage of development (A), but they elongate later in embryogenesis and appear rectangular at the L1 stage as viewed in an image of the posterior region (B). Identified cells are labeled in white. Hypodermal cells remain round or square shaped in *rhgf-2(gk216)* mutants indicating embryonic cell shape changes did not occur properly in the mutants (C). The control strain, which has an embryonic AJM-1::GFP expression pattern similar to wild type, is *jcIs1[ajm-1::gfp; pRF4; C45D3]; him-5*. The genotype of the *rhgf-2* strain is *rhgf-2(gk216); jcIs1*. Scale bars are 25 µm.

Time-lapse video analysis revealed that *rhgf-2(gk216)* mutants arrested elongation early in the elongation process at the 1.5 fold stage of embryogenesis (Video S1). Cell differentiation did occur beyond the 1.5 fold stage as the intestine and a pumping pharynx were visible after hatching and larvae exhibited minor muscle movements. Since hypodermal cells provide the force for the initial stages of elongation [37] the apical cell junction marker AJM-1::GFP [38]–[40] was used to examine hypodermal cell morphology in *rhgf-2(gk216)* mutants. Note that hypodermis is the common term for the *C. elegans* epidermis. Hypodermal seam cells, which in wild-type animals elongate during embryogenesis and appear rectangular at larval stage one (L1), were more round or square shaped in the *rhgf-2(gk216)* mutants at the same stage (Fig. 6). This indicated that the *gk216* elongation defect may result from a lack of proper hypodermal cell shape changes that are required during embryogenesis.

Rescue of the *rhgf-2(gk216)* developmental arrest and severe Dpy phenotype was obtained in *rhgf-2(gk216)* animals containing transgenic DNA spanning the *rhgf-2* genomic region (Fig. 3), however, all of the rescued strains laid fewer eggs than wild type and some mutants containing the rescuing transgene exhibited partial rescue (Table 1). Partially rescued animals grew to the adult stage, but maintained a moderate Dpy phenotype, possibly due to variations in the frequency of spontaneous loss of the extrachromosomal array at some cell divisions during development. These transgenic rescue experiments indicated the deletion within the *rhgf-2* gene is most likely responsible for the phenotypes observed in *gk216* animals. These experiments also questioned the role of the *rhgf-2l* 5′ region as smaller DNA fragments encoding only RHGF-2s (short isoform) were sufficient to rescue the developmental arrest and Dpy phenotypes in most *rhgf-2(gk216)* animals (Fig. 3; Table 1). Therefore, *rhgf-2* likely encodes at least two mRNA transcripts (l and s, for long and short) each with their own promoter (Fig. 3). If the RHGF-2 N-terminal region acted as an autoinhibitory domain, similar to RhoGEFs such as ASEF, Dbl and Vav, it could explain why the N-terminus is not required for complete rescue of the *rhgf-2* phenotype [41]–[44]. However, the RHGF-2 N-terminal region has no sequence similarity to the autoinhibitory domains of these RhoGEFs. *rhgf-2* may be another example of a gene, such as *unc-5*, which does not require the most 5′ coding exons or the regulatory region 5′ of the first exon, for rescue of the mutant phenotype [45].

**Table 1:** Categorization of progeny from rescued *rhgf-2(gk216)* transgenic lines compared to wild type.

Strain*	Transgene Encodes	Unhatched Eggs (%)	L1 Arrested (%)	Dpy Adult§ (%)	nonDpy Adult# (%)	Eggs Laid/Worm/24 hrs† (n)
N2	-	0 (0)	0 (0)	0 (0)	4146 (100)	138 (30)
gk216;Ex102-A	rhgf-2l	12 (1)	526 (33)	53 (3)	1002 (63)	59 (27)
gk216;Ex102-D	rhgf-2l	22 (6)	181 (51)	44 (12)	109 (31)	30 (12)
gk216;Ex113-A	rhgf-2s	19 (2)	378 (46)	59 (7)	373 (45)	69 (12)
gk216;Ex113-B	rhgf-2s	7 (1)	216 (39)	88 (16)	244 (45)	23 (24)

*Independent extrachromosomal transgenic lines are identified by different single letter designations at the end of the strain names.

§ Partially rescued animals (with respect to the transgenic lines and not N2).

# Fully rescued animals (with respect to the transgenic lines only).

† Young adults (animals were less than 12 hours beyond the L4 stage) laid eggs over a 24-hour period and the progeny were categorized as they developed over four days. n = number of animals plated for each strain.

### RHGF-2::GFP is expressed in the nervous system

**Figure 7:**
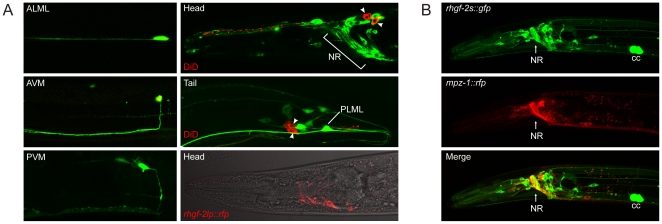
RHGF-2 expression is restricted to the nervous system and co-localizes with MPZ-1. *A*. A functional *rhgf-2s::gfp* transgene (Fig. 3) was used to examine *rhgf-2* expression in a wild-type background. Animals were also stained with DiD (red) to label the ciliated sensory neurons, none of which expressed RHGF-2s::GFP (green). Two of the twelve stained sensory neurons are visible in the head plane of view, while two of the four in the tail are visible (arrowheads). RHGF-2s::GFP localization is shown in neurons in the head and the tail as well as the ALM, AVM, PVM and PLM mechanosensory neurons. The last panel shows RHGF-2lp::RFP fluorescence (red) in a small number of head neurons. RHGF-2lp::RFP is also expressed in two neurons in the tail (not shown). *B*. RHGF-2s co-localizes with MPZ-1 in the axons of the nerve ring (NR). Anterior is to the left and ventral is down in all images. The coelomocytes (cc) fluoresce as a result of the co-transformation marker.

To investigate RHGF-2 expression in *C. elegans*, a *rhgf-2s::gfp* construct was assembled containing *rhgf-2* genomic DNA starting from 5.7 kb upstream of the predicted *rhgf-2s* start ATG through to the last amino acid codon, which was fused to GFP coding sequence with a 3′ UTR from *let-858* (Fig. 3). The *rhgf-2s::gfp* construct was functional since it rescued the *rhgf-2(gk216)* early developmental arrest and severe Dpy phenotypes. Somewhat surprisingly, considering the developmental arrest phenotype of *rhgf-2(gk216)* animals, GFP fluorescence in *rhgzzzf-2s::gfp* transgenic animals appeared exclusively neuronal during development from about the 1.5 fold stage of embryogenesis through to the adult (Fig. 7). No obvious RHGF-2s::GFP fluorescence was visible in the earlier stages of embryogenesis. Many, but not all, of the 302 *C. elegans* hermaphrodite neurons expressed GFP in the transgenic animals examined (Fig. 7). Several cell bodies in head and tail ganglia and many neuronal processes in the nerve ring and ventral cord were visible, with the GFP fluorescence for most cells distributed diffusely throughout the cell, although there were instances of more punctate fluorescence within some cell bodies and processes, particularly in the ventral cord. Neurons that were positively identified in late larval/adult animals based on their position and morphology were the ALM, AVM, PLM and PVM mechanosensory neurons and the BDUs (Fig. 7). Expression from the ciliated sensory neurons, most ventral cord motor neurons and neurons with cell bodies in the mid-body of the animal such as the CANs, PDEs and SDQs, for example, was notably absent from transgenic animals observed at late larval and adult stages.

Expression driven by the 5′ regulatory regions of the full length *rhgf-2l* transcript was examined using a tagRFP transcriptional fusion reporter (*rhgf-2lp::rfp*; Fig. 3). Fluorescence from *rhgf-2lp::rfp* transgenic animals was also restricted to the nervous system, but to a smaller subset of neurons that partially overlapped with *rhgf-2s::gfp* expression (Fig. 7A).

If RHGF-2 and the MPZ-1 scaffold protein interact *in vivo* we would expect to see co-localization of these proteins in at least some cells of the animal. Previous analysis revealed that MPZ-1 is expressed in a large portion of the nervous system as well as the muscles of the body wall and the vulva [27]. Transgenic lines containing the rescuing *rhgf-2::gfp* transgene and an *mpz-1::tagrfp* transgene driven by the MPZ-1 neural promoter [27] were examined for RHGF-2 and MPZ-1 co-localization. As expected, RHGF-2::GFP was expressed in many of the same neurons expressing MPZ-1::tagRFP (Fig. 7B; data not shown). MPZ-1::tagRFP was most strongly localized to the axons of the nerve ring where RHGF-2::GFP was also highly distributed (Fig. 7B). The co-localization of RHGF-2 and MPZ-1 in the nerve ring is consistent with the hypothesis that these proteins function together in a neuronal signaling pathway in at least a subset of the neurons in the nervous system.

### RHGF-2 expression alters mammalian neuron morphology and *C. elegans* locomotion

**Figure 8:**
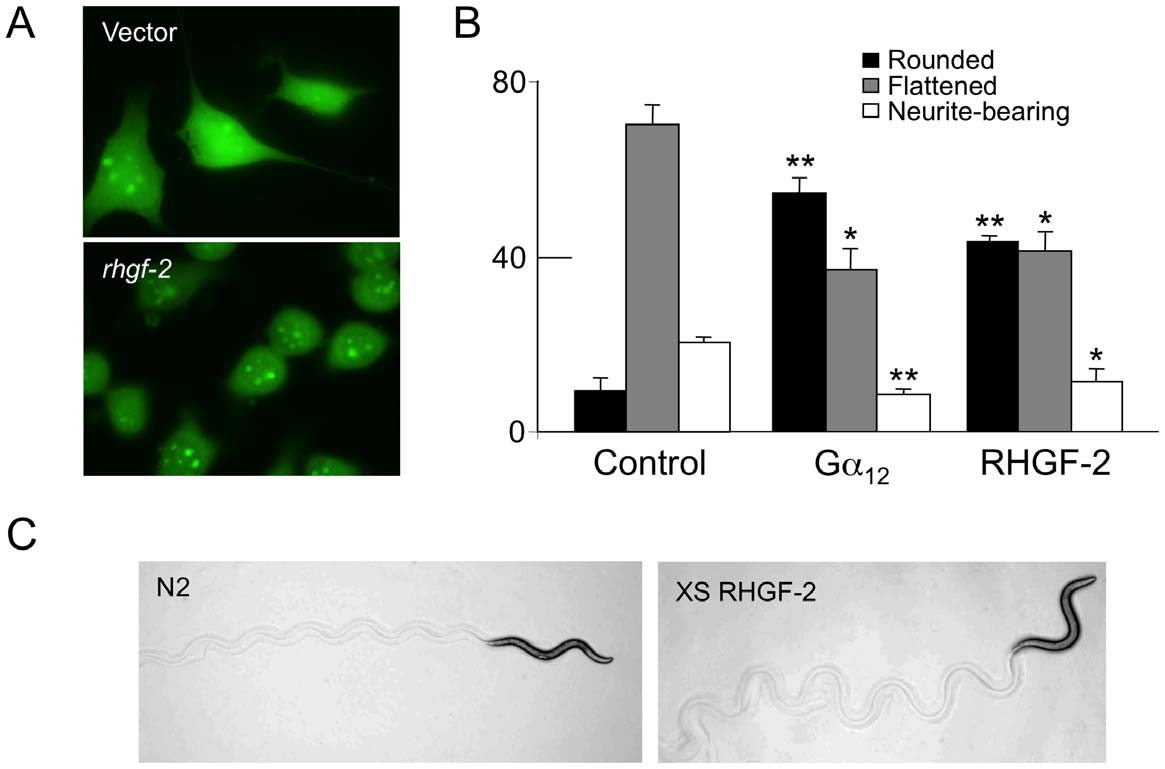
RHGF-2 expression alters the morphology of N1E-115 cells and regulates *C. elegans* movement. *A & B*. N1E-115 cells were transiently transfected with FLAG vector, DNA encoding FLAG-tagged RHGF-2s or DNA encoding constitutively active Gα_12_ that served as a positive control. After 4–6 hours, cells were transferred to serum-free DMEM to induce morphological differentiation. After 48 hours, the cells were fixed and visualized with anti-FLAG and FITC-coupled secondary antibody. The percentage of rounded, flattened and neurite-bearing transfected cells (with neurites the length of at least twice the cell body diameter) averaged from three independent experiments is indicated in the graph. At least 200 transfected cells (green) were examined in each experiment. Error bars represent SEM. **p<0.001 and *p<0.01 in comparison to the control cells using the Student's *t*-test. *C*. RHGF-2s overexpression from an extrachromosomal array alters *C. elegans* locomotion. The track left in the bacterial lawn by an N2 animal has a characteristic sinusoidal wave pattern (left panel). An animal overexpressing RHGF-2s (XS RHGF-2; *Ex115[rhgf-2s::gfp] rhgf-2(gk216)*) moves with exaggerated body bends as revealed by the track with a higher amplitude wave compared to N2 (right panel).

Rho family GTPases play important roles in neuronal development and the control of neuron morphology [29]. For example, G protein coupled receptor agonists such as serum-borne lysophosphatidic acid trigger rapid growth cone collapse, retraction of developing neurites, and transient rounding of the cell body as the result of RhoA activation in neuronal cell lines [46]–[48]. To examine RHGF-2 effects on neuron morphology we transiently expressed FLAG::RHGF-2 or FLAG alone in N1E-115 cells. FLAG::RHGF-2 expression in N1E-115 cells induced cell rounding and inhibited neurite outgrowth to a similar extent as activated Gα_12_ (Fig. 8). Activated Gα_12_, like LPA, was previously demonstrated to induce cell rounding and neurite retraction through RhoA mediated pathways [49], [50].

Additional observations were consistent with RHGF-2 functioning in *C. elegans* neurons. Constitutive Rho GTPase activity in *C. elegans* cholinergic motor neurons results in hyperactive movement and exaggerated body bend (“loopy”) phenotypes due to Rho-mediated increases in motor neuron acetylcholine release [3]. Similarly, transgenic animals that overexpressed Rho-specific RHGF-2s or RHGF-2l from an extrachromosomal array, in either a wild-type or *rhgf-2(gk216)* background moved in a loopy manner in comparison to wild type (Fig. 8C). Additional analysis of RHGF-2 function in the nervous system is required to further define its role in the regulation of *C. elegans* locomotion.

## Discussion

*C. elegans* RHO-1 is required at the earliest stages of development for cytokinesis and cell polarity [51]–[53] and in later stages of development for epidermal P cell migrations, embryo elongation and myosin thick filament organization in muscle cells [54]–[56]. In adult animals RHO-1 signaling plays non-developmental roles in the modulation of locomotion, pharyngeal pumping, egg laying, defecation cycling, and cell morphology [3], [57]. RHO-1 is likely activated by multiple RhoGEF proteins operating in different pathways and in this study we focused on the characterization of the *C. elegans* RHGF-2 RhoGEF.

Our analysis of the *rhgf-2(gk216)* deletion mutant revealed RHGF-2 is required early in development for embryo elongation. The role of RHGF-2 in elongation during embryogenesis, however, is not obvious, particularly since *rhgf-2* expression was only observed in a portion of the nervous system, from the middle stages of embryogenesis onward. Neuroblasts, however, do play a role in epidermal morphogenesis by acting as substrates for epidermal cell movements early in *C. elegans* development. The *C. elegans* Eph receptor tyrosine kinase VAB-1 (variable abnormal) and its ephrin ligands (EFN-1 to 3; eph(f)rin) are required in neuroblasts for epidermal morphogenesis during embryogenesis [58]–[62]. Interestingly, another positive in our yeast two-hybrid screening with MPZ-1 was VAB-1 (unpublished data), however, since the early *vab-1* and *efn* phenotypes of incomplete ventral enclosure are distinct from the *rhgf-2* elongation defects occurring after enclosure, it does not appear that RHGF-2 functions similarly to VAB-1 and the ephrins in epidermal morphogenesis.

Another possibility is that RHGF-2 is expressed directly in epidermal cells during embryogenesis, but RHGF-2::GFP fluorescence is just not detected in our transgenic lines. RHGF-2::GFP may be more weakly expressed outside the nervous system or perhaps there is a C-terminally truncated RHGF-2 isoform we are not aware of that is not visible with our current positioning of the GFP tag in the *rhgf-2* gene. *C. elegans* embryos elongate as a result of circumferential actin based contractions within epidermal cells and the RHO-1 GTPase target of RHGF-2 and RHO-1 effectors play defined roles in the regulation of these contractions [37], [59], [60]. Genetic and biochemical evidence indicates Rho activates Rho-kinase (LET-502 in *C. elegans*; lethal) to inhibit smooth muscle myosin phosphatase (MEL-11; maternal effect lethal) resulting in increased myosin phosphorylation and actin contraction [63], [64]. Additional methods to examine RHGF-2 distribution should help determine if RHGF-2 functions in *C. elegans* epidermal cells with respect to embryo elongation. It is intriguing that the loss of the zebrafish RHGF-2 homolog, Syx, also results in shortened embryos that arrest development [24]. The mechanism of Syx function in zebrafish development is not known.

In addition to the role in embryo elongation RHGF-2 likely works with RHO-1 in a post-developmental role as well. We observed that transgenic animals overexpressing RHGF-2 move with exaggerated body bends, which is a locomotory phenotype similar to the activated RHO-1 phenotype [3] and suggests that RHGF-2 activates RHO-1 to regulate *C. elegans* locomotion. RHO-1 modulates locomotion rate through diacylglycerol kinase (DGK) dependent and DGK independent pathways, which alter cholinergic neurotransmitter signaling [3], [65]. The RhoGEFs RHGF-1 and UNC-73 also play roles in controlling locomotion rate and it will be interesting to examine the relationship between these *C. elegans* RhoGEFs [15], [65]–[67].

We identified RHGF-2 as an MPZ-1 interacting protein based on yeast two-hybrid screening, *in vitro* protein-protein interactions, co-immunoprecipitations and *in vivo* co-localization. PDZ domain-containing scaffolding proteins like MPZ-1 are thought to increase the efficiency of signal transduction pathways by localizing interacting pathway members to specific regions of the cell, such as tight junctions or synapses [30], [68]. Interactions with PDZ domains are most often mediated through short C-terminal PDZ binding motifs [30]. Bioinformatic analysis of human proteins revealed approximately 40% of RhoGEFs and a large number of RhoGAPs (GTPase activating proteins) contain a putative PDZ binding motif indicating the importance of Rho GTPase signaling to the function of PDZ scaffold-mediated signaling complexes [69].

In mammalian neurons the MPZ-1 homolog MUPP1 interacts with serotonin and GABA_B_ neurotransmitter receptors, olfactory sensory receptors as well as SynGAP, a synapse-specific RasGAP, and Ca^2+^/calmodulin-dependent kinase (CaMKII) to regulate neuronal signaling and dendritic spine morphology [7], [70]–[73]. MUPP1 also binds the Tech and Kalirin-7 RhoGEFs, which are the homologs of *C. elegans* RHGF-2 and UNC-73, respectively [26], [74]. Kalirin-7 localization in the post-synaptic density is required for serotonin induced changes in spine morphology but the precise relationship between Rho GTPase pathways and signaling mediated by MUPP1 complexes is not known [7]. Examination of MUPP1 interactions in non-neuronal cells identified several additional interacting proteins, which indicate MUPP1 potentially integrates multiple signal transduction pathways and can regulate tight junction integrity for epithelial function [75]–[85].

**Figure 9:**
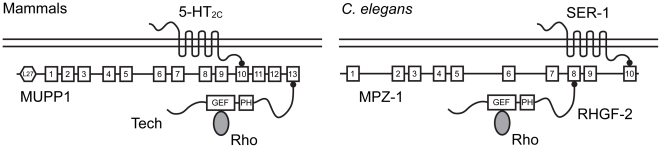
MUPP1/MPZ-1 scaffolding protein interactions are conserved between mammals and *C. elegans*. A schematic representation of PDZ domain interactions conserved between mammals (left) and *C. elegans* (right). Tech interacts with MUPP1 PDZ domains 10 and 13, but for clarity only the interaction with PDZ 13 is shown. Similarly, RHGF-2 also interacts with less affinity to MPZ-1 PDZ domains 9 and 10. Other documented PDZ domain interactions that may not be conserved are described in the text. L27 is a domain originally identified in the receptor targeting proteins LIN-2 and LIN-7. The numbered boxes indicate the PDZ domains in MUPP1 and MPZ-1. The small black circles represent PDZ-binding domains.

The functions of MUPP1 in neuronal signaling at the synapse are consistent with our observation of RHGF-2 involvement in the regulation of locomotion. In *C. elegans*, MPZ-1 binds the phosphatase and tensin (PTEN) ortholog DAF-18 and the arrestin ARR-1 to regulate longevity through DAF-2 insulin-like growth factor receptor signaling [86]. MPZ-1 also interacts with the SER-1 serotonin receptor and *mpz-1* RNAi revealed MPZ-1 is required in vulval muscles to facilitate serotonin stimulated egg laying, but MPZ-1's role in the *C. elegans* nervous system, where it is also expressed, has not been examined [27]. In conclusion, there are at least two interactions involving the MUPP1/MPZ-1 scaffolding proteins that are conserved between mammals and *C. elegans*. MPZ-1 binds the RHGF-2 RhoGEF protein and the SER-1 serotonin receptor in *C. elegans*, while MUPP1 interacts with the RhoGEF Tech and the 5-HT_2C_ serotonin receptor in mammals (Fig. 9). Our *C. elegans* studies provide insight into the *in vivo* function of the RHGF-2/Tech family of RhoGEFs and provide a framework for the additional dissection of the signaling pathways involving this important RhoGEF family and the MPZ-1/MUPP1 scaffolding proteins.

## Materials and Methods

### Strains and cell lines

*C. elegans* strains were maintained with *E. coli* OP50 at 21°C on plates containing standard nematode growth media. The strains N2 Bristol (wild type) and VC455 *rhgf-2(gk216)*/*mIn1*[*mIs14 dpy-10(e128)*] II, which was produced by the *C. elegans* Gene Knockout Consortium, were obtained from the Caenorhabditis Genetics Center (University of Minnesota, Minneapolis). *rhgf-2(gk216)* was backcrossed five times to wild type and placed over the *mIn1* balancer to produce the strain XS245 rhgf-2(gk216)*5/*mIn1*. The backcrossed version of *rhgf-2(gk216)* was used in all experiments described in this paper. *jcIs1*[pJS191(*ajm-1::GFP*); pRF4(*rol-6 (su1006dm*)); C45D3(*unc29(*+*))*]; *him-5(e1490)* was kindly provided by Joe Culotti (S. Lunenfeld Research Institute, Toronto, Canada). *jcIs1* was produced by Jeff Simske [39]. Standard genetic methods were used to create the strain rhgf-2(gk216)*5/mIn1; jcIs1 for analysis. HEK293T and N1E-115 cells were obtained from the American Type Culture Collection (ATCC) (Manassas, VA).

### Yeast two-hybrid screening

An *mpz-1* cDNA encoding PDZ domains 8–10 was cloned into the bait vector pEG202 (Origene Technologies Inc., Rockville, MD). This clone was used to screen approximately 2×10^7^ transformants from a *C. elegans* adult cDNA library (Origene Technologies Inc., Rockville, MD) following the manufacturer's instructions. Over 100 positive clones were picked and saved for analysis. Of the 40 positive clones for which sequence information was obtained, two of the clones were identified as *rhgf-2*. Similar yeast two-hybrid screens performed using *mpz-1* clones encoding PDZ domains 1–4 or 5–7 did not yield any *rhgf-2* positives.

### rhgf-2 cDNA analysis

An oligo dT-primed cDNA library pool was created using Superscript II (Invitrogen, Carlsbad, CA) reverse transcriptase and mRNA purified from mixed stage *C. elegans* N2 animals. Identical *rhgf-2* cDNAs were amplified by PCR from the cDNA pool and another mixed stage *C. elegans* library (Origene Technologies) using a forward primer, RS521, to predicted gene TO8H4.2 and a reverse primer, RS523, to *rhgf-2*. Table S1 lists the primer sequences. The sequence of a portion of the new *rhgf-2* cDNA is printed below. The unshaded portion indicates the 3′end of exon 3 (previously designed as T08H4.2 exon 3 sequence) while the shaded portion indicates the 5′ end of the new exon 4 (the previous designation for *rhgf-2* exon one began with the ATG in white letters).

…GGGAGCACGCTTCCTGGCTGGCCAGAGAATTTTCGTTAGAGGACGAGGGAATATGAAAGTGGTGAGACACACACGGGCCACACACAGTATGGACGAAAGTAACGAGCGAGCAACGAGAAAAATGAGCGCTGAAGCTG…

The intron sequences in the genomic DNA immediately adjacent exon 3 and exon 4 (not shown in the cDNA sequence above) match consensus splice donor and acceptor sequences, respectively, indicating the cDNA sequence likely represents a true mRNA and not a fusion artifact generated in the production of the cDNAs. Figure S1 contains the full sequence of the *rhgf-2l* cDNA and the predicted protein. Our *rhgf-2* cDNA analysis also revealed evidence for the existence of a shorter transcript, *rhgf-2s*, as diagramed in Figure 3. A cDNA beginning with a trans-spliced SL1 leader sequence on the 5′ end of *rhgf-2l* exon four (the shaded region in the sequence above) was identified by PCR from the Origene mixed stage cDNA library using a forward primer, UT131, specific to the vector sequence and a reverse primer, RS524, which hybridized to *rhgf-2*.

### Generation of transgenic lines

*rhgf-2* and *mpz-1* genomic DNA fragments were made by PCR using either iProof (Bio-Rad, Hercules, CA) or PCR Extender (Fisher Scientific, Pittsburgh, PA) high fidelity DNA polymerases. Genomic DNA fragments were produced in triplicate, confirmed by restriction enzyme mapping and mixed together before injecting into animals. Overlapping genomic DNA fragments were mixed together in equimolar concentrations. *rhgf-2* and *mpz-1* DNAs tagged with either GFP or tagRFP were produced by the technique of PCR fusion [87]. Each tag was followed by the *let-858* 3′ UTR (approximately 450 bp). The *rhgf-2* DNA fragments generated in this study are indicated in Figure 3. The following is a list of the extrachomosomal arrays, the primers used to generate the DNA fragments in the arrays and in brackets, the sizes of the included 5′ regulatory regions upstream of the ATG: *Ex73*, RS796/RS797 (3.3 kb); *Ex102* RS796/RS945 (3.4 kb); *Ex104*, RS974/UT19 and UT20/RS945 (6.4 kb); Ex110, UT20/UT28 (5.7 kb); Ex113 UT25/RS945 (3 kb). Five genomic DNA fragments spanning the *mpz-1* gene, including 3 kb of 5′ regulatory region, were generated with the most 3′ fragment fused to tagRFP (with a *let-858* 3′ UTR) after the last *mpz-1* amino acid codon. The primers used to generate the five *mpz-1* genomic DNA fragments were UT78/UT79, UT80/UT81, UT82/UT83, UT84/UT85 and UT89/UT93. Each *mpz-1* fragment is between 7 and 10 kb in length with 1 to 1.5 kb of overlap between fragments. Table S1 lists primer sequence information.

Standard microinjection techniques were used to generate stable transgenic *C. elegans* lines carrying extrachromosomal DNA arrays [88]. Overlapping genomic DNA fragments in a DNA injection mix were assumed to undergo homologous recombination in the animal [89]. From 5 to 75 ng/µl of *rhgf-2* or *mpz-1* DNA was mixed with 50 ng/µl of cotransformation marker *unc-122p::gfp* (coelomocyte specific promoter) and 100 ng/µl of herring sperm DNA for injection. An exception to the above is that 50 ng/µl of the co-transformation marker pRF4 [*rol-6(su1006dm)*] was used with the *rhgf-2p::rfp* construct instead of the coelomocyte marker. *rhgf-2* DNA mixes were injected into rhgf-2(gk216)/mIn1*5 or N2 animals, while the *mpz-1* DNA was only injected into N2. Progeny were screened for stable expression of the extrachromosomal array and homozygous *rhgf-2(gk216)* lines containing the array were established. If the DNA was injected into N2 animals the extrachromosomal array was crossed into the *rhgf-2(gk216)* background. At least three independent transgenic lines for each extrachromosomal array were examined. To examine co-localization of *rhgf-2lp::rfp* and *rhgf-2s::gfp* expression, the *uxEx110[rhgf-2s::gfp]* extrachromosomal array was crossed into the *uxEx73[rhgf-2lp::rfp]* transgenic line.

### GST pull down assays

MPZ-1 PDZ domain GST fusion proteins (15 ug) purified from bacteria were incubated for 4 hours at 4°C with HEK293T lysates (80 ug) from cells transfected with DNA encoding FLAG::RHGF-2s. Glutathione-sepharose beads were added, and the incubation was continued for an additional 60 min. Beads were collected by centrifugation and washed four times with 0.5 ml of binding buffer to remove unbound proteins. Bound proteins were eluted from the beads, separated by SDS/PAGE, and analyzed by Western blotting with FLAG antibody. Radiolabelled RHGF-2s, with or without the PDZ-binding motif, was prepared by *in vitro* transcription/translation using the TNT-T7 Quick Coupled rabbit reticulocyte system (Promega, Madison, WI) and 20 µCi [^35^S] methionine (1000 Ci/mmol). [^35^S]-labeled RHGF-2s was incubated for 4 hours with glutathione sepharose beads previously saturated with GST::MPZ-1(PDZ8-10) fusion protein. Beads were washed four times and bound proteins were resolved by SDS-PAGE and visualized by autoradiography.

Binding of RHGF-2s to Rho family GTPases was performed essentially as described [90]. GST fusions were expressed in *E. coli* and induced with 0.3 mM IPTG for 2 hours at 37°C. Cells were sonicated in 20 mM HEPES (pH 7.9), 100 mM KCl, 0.5 mM EDTA, 1 mM dithiothreitol, 1 mM PMSF, and protease inhibitors. Fusion proteins were purified from lysates on glutathione-sepharose beads. FLAG tagged *rhgf-2s* was transfected into HEK293T cells. Forty-eight hours after transfection, cells were lysed in 150 mM NaCl, 5 mM MgCl, 0.5% Nonidet P-40, and protease inhibitors. 200 µg of cell lysate was incubated with 20 µg of GST fusion protein for 3 hours at 4°C. Bound proteins were eluted from the beads, separated by SDS/PAGE, and analyzed by Western blotting with FLAG antibody.

### Co-immunoprecipitation

A cDNA encoding RHGF-2s was subcloned into p3×FLAG-CMV-7.1 (Sigma, St. Louis, MO). *rho-1*, *cdc-42* or *ced-10* sequence in frame with GST coding sequence was subcloned into pcDNA3.1 (Invitrogen). 5 µg of *flag::rhgf-2* DNA was transiently cotransfected together with 5 µg of *gst::rho-1*, *gst::ced-10* or *gst::cdc-42* into HEK293T cells. Cells were harvested 48 hours post-transfection after washing with cold phosphate buffered saline (PBS) and lysed using 500 µl of lysis buffer (0.5% NP-40, 150 mM NaCl, 50 mM Tris (pH 8.0), 1 mM Na_3_VO_4_, 1 mM PMSF and protease inhibitors). Lysates were incubated with anti-GST antibody (Sigma) for 3 hours at 4°C. The immunocomplexes were recovered using Protein A/G agarose beads. RHGF-2s that co-precipitated with the small GTPases was visualized by immunoblotting with anti-FLAG antibody following SDS-PAGE.

### *In vitro* guanine nucleotide exchange assay

GST-fused *C. elegans* small GTPases were purified from *E. coli* as described above. GDP-loaded GST::RHO-1, GST::CED-10 or GST::CDC-42 (1 µM) was incubated at 30°C in exchange buffer (50 mM HEPES pH 7.5, 100 mM KCl, 2 mM MgCl_2_, 1 mM DTT, 0.1 nM [^35^S]GTPγS) in the presence or absence of FLAG::RHGF-2s that was immunoprecipitated from transfected HEK293T cells. After a 15 min incubation the reaction mixtures were filtered through nitrocellulose disks, which were rinsed with stop buffer (50 mM HEPES pH 7.5, 10 mM MgCl_2_). The amount of [^35^S]GTPγS bound to GTPase on the filter was determined as the number of counts per minute measured using a liquid scintillation counter.

### SRE-Luciferase Assay

HEK293T cells (6×10^4^ cells per well) were plated onto 24-well plates one day before transfection. Cells were cotransfected with SRE-luciferase reporter plasmid (0.1 µg), pCMVßgal (0.1 µg), and the indicated cDNAs. Cells were cultured in the presence of 10% FBS for 5 hours, washed twice with PBS, then serum-starved for 24 hours. Cells were then lysed using reporter lysis buffer (Promega) and luciferase activities in the cell extracts were measured according to the manufacturer's instructions (Promega). Total amounts of transfected DNA were kept constant among wells by supplementing with empty pCDNA3.1 vector DNA. ß-galactosidase activities present in each sample were assayed by a colorimetric method, and used to normalize for transfection efficiency.

### Immunocytochemistry

NIE-115 cells were cultured at 37°C in the presence of 5% CO_2_ in DMEM supplemented with 10% fetal bovine serum (FBS) and 1% penicillin/streptomycin. Cells were plated on coverslips placed in six-well plates and cultured for 24 hours before transfection. A total of 1 µg of expression plasmids was transfected into cells using Lipofectamine 2000 (Invitrogen). 12 hours after transfection the medium was replaced with serum free DMEM to induce morphological differentiation. Transfected cells were washed twice with PBS, fixed with HistoChoice tissue fixative (Amresco, Solon, OH) for 30 min and permeabilized with 0.1% Triton X-100 in PBS for 5 min. Cells were washed three times with PBS and blocked with 5% heat inactivated goat serum in PBS for 30 min. Cells were immunostained with mouse polyclonal anti-FLAG antibody (1∶10 dilution) for 1 hour and washed three times with PBS. After incubation with FITC secondary antibodies for 30 min, cells were washed three times with PBS and mounted on cover slides. Changes in cell shape were monitored using a Zeiss Axiophot microscope at 40× magnification with a GFP filter set. Cells were scored as rounded, flattened, or neurite-bearing. Neurites had to reach a length of at least twice the cell body diameter to be counted. For each transfection, the percentage of rounded, flattened, and neurite-bearing cells was calculated from at least 200 green cells. An average percentage was calculated from at least three independent experiments.

### Confocal Microscopy

Worms were immobilized with 30 mg/ml BDM (2,3-butanedione monoxime; Sigma) in M9 and mounted on 2% agarose pads for examination by epifluorescence. Animals were stained with 1,1′-dioctadecyl-3,3,3′,3′-tetramethylindodicarbocyanine perchlorate (DiD) as described previously [91]. Images were obtained with an Olympus Fluoview 300/IX70 confocal microscope and Olympus Fluoview 5.0 software.

### Video Microscopy

Animals were picked to standard nematode growth media plates with bacteria and left to lay eggs for about three hours at room temperature. Eggs were mounted on a 5% agarose pad with M9 for imaging at room temperature. All four *rhgf-2(gk216)* embryos that were imaged arrested elongation at the 1.5 fold stage of embryogenesis. Video images were captured with a Photometrics CoolSNAP HQ^2^ camera mounted on an Olympus IX81 microscope with a PlanApo N 60×/1.42 oil objective and processed using Slidebook software (Intelligent Imaging Innovations, CO).

## Supporting Information

Figure S1 **The** ***rhgf-2l*** **cDNA and translated protein sequences.** Exons are indicated by alternate yellow and orange highlighting. The nucleotides deleted in *gk216* are indicated in bold. The *rhgf-2* exon sequence identified in this study is boxed. The RhoGEF domain is located between amino acids 280–467 and the PH domain from amino acids 525–628.
(DOCX)

Video S1 ***rhgf-2(gk216)*** **mutants do not elongate past the 1.5 fold stage of embryogenesis.** A time-lapse video of *rhgf-2(gk216)* (top) and *rhgf-2(gk216)/mIn1* (bottom) embryonic development. The *gk216* embryo arrests development at the 1.5 fold stage, although it is still capable of small movements. The control *gk216* heterozygous embryo of a comparable age continues through to the three-fold stage of embryogenesis before the end of the video. Images were captured every minute over 6.5 hours.
(MOV)

Table S1 **DNA Oligonucleotide Primer List.** This table lists the oligonucleotide primers used in the PCR reactions performed in this study.
(DOC)
