# Supplementary material for: RHGF-2 Is an Essential Rho-1 Specific RhoGEF that binds to the Multi-PDZ Domain Scaffold Protein MPZ-1 in Caenorhabditis elegans
Source: PLoS One. 2012 Feb 20;7(2):e31499. doi: 10.1371/journal.pone.0031499 (PMC3282746; doi:10.1371/journal.pone.0031499)
Supplement: Figure S1 — The rhgf-2l cDNA and translated protein sequences. Exons are indicated by alternate yellow and orange highlighting. The nucleotides deleted in gk216 are indicated in bold. The rhgf-2 exon sequence identified in this study is boxed. The RhoGEF domain is located between amino acids 280–467 and the PH domain from amino acids 525–628. (DOCX) [file pone.0031499.s001.docx]

1 M S S S V G H A R S R R P S L H N D W T

1 ATGAGCAGTAGTGTTGGACATGCTCGAAGCAGGAGACCATCTCTGCACAATGATTGGACA

21 N E H A Q R N A L D N R I H H I D I D P

61 AATGAGCACGCTCAGAGGAATGCATTAGATAATCGCATTCATCACATTGACATCGATCCA

41 P Q I N Y F I A V F K D L D D R E K D S

121 CCGCAGATCAACTATTTCATCGCAGTTTTTAAGGATTTGGACGATCGAGAGAAAGATTCC

61 V E H I R F D C N Q L I Y E V F E P F L

181 GTCGAGCATATTAGATTCGATTGTAATCAACTGATTTATGAAGTTTTTGAACCGTTCCTC

81 R I R G L T I N D V E F F L E K S S T P

241 CGTATCCGAGGCCTTACCATCAATGATGTGGAATTCTTCCTCGAGAAGAGCTCAACCCCG

101 I P E N S G A R F L A G Q R I F V R G R

301 ATTCCAGAAAACTCGGGAGCACGCTTCCTGGCTGGCCAGAGAATTTTCGTTAGAGGACGA

121 G N M K V V R H T R A T H S M D E S N E

361 GGGAATATGAAAGTGGTGAGACACACACGGGCCACACACAGTATGGACGAAAGTAACGAG

141 R A T R K M S A E A V S R K S S F V N S

421 CGAGCAACGAGAAAAATGAGCGCTGAAGCTGTTTCGAGGAAGTCATCGTTTGTCAATAGC

161 R I L N R G S R N H P G L N G S D D A E

481 AGAATT**CTCAACCGTGGTTCTCGAAATCATCCAGGATTGAATGGAAGTGATGACGCCGAA**

181 C R S N D S S L S V R D S W Q Y D E P S

541 **TGTCGGAGCAATGACAGCTCGTTGAGTGTACGCGACAGTTGGCAATACGACGAGCCATCG**

201 C S E V P D D G G T R R A R S V T S S R

601 **TGTTCCGAAGTTCCAGACGACGGTGGC**ACCCGACGCGCCCGATCAGTAACCTCTTCGCGT

221 I S L F F G K E T G K I E K E M F R I L

661 ATTTCTTTGTTCTTCGGCAAAGAAACCGGGAAAATCGAAAAGGAAATGTTTAGAATATTG

241 N Q K K E D T D L E Y P T Y K L E K D W

721 AATCAAAAGAAAGAGGACACCGATCTAGAGTACCCGACGTATAAGCTGGAGAAAGATTGG

261 R E I V H N H E S L S D K S I K Q Q E A

781 CGGGAGATTGTGCATAATCACGAGAGTTTGTCGGACAAGAGTATCAAGCAACAAGAAGCT

281 I W E I V T T E F R Y I K L L R Y L S N

841 ATTTGGGAGATTGTGACGACTGAATTTAGATATATCAAGCTTCTCCGTTACCTGTCGAAT

301 L S F Y L T D L Q N C G F L K D I E N R

901 TTGTCCTTTTACCTTACGGATCTTCAAAATTGCGGGTTTTTGAAAGATATCGAAAATCGT

321 L V F F N F V T L F N V N Y D S L W L Q

961 CTAGTGTTCTTCAATTTCGTTACACTTTTCAATGTGAATTACGACTCCCTGTGGCTCCAG

341 S I E P I L A K S R E T G E P L D V N Y

1021 TCGATTGAGCCAATCCTTGCGAAATCTCGTGAAACTGGAGAGCCCCTTGACGTGAACTAC

361 L Q N G F R D I E N W S R C Y T N F H L

1081 CTGCAAAATGGGTTCAGAGACATTGAGAACTGGTCGAGGTGCTACACGAACTTCCATCTG

381 A H S D S L K H I Q K K L K E S E N F R

1141 GCTCACAGTGATTCACTGAAACATATTCAGAAGAAGCTAAAAGAGAGCGAGAATTTTAGG

401 D F V T W A E A Q E N L D R Q K L I D T

1201 GATTTTGTGACGTGGGCCGAAGCGCAGGAAAACTTGGATCGTCAGAAGCTGATCGACACG

421 F S V P M Q R L T R Y N L L L K A V L K

1261 TTTTCGGTTCCAATGCAGCGGTTAACCAGATACAATTTATTGCTGAAAGCTGTCCTGAAA

441 V T T D E N E R E M I S N L V D C A E S

1321 GTCACCACCGACGAGAACGAACGAGAAATGATCAGCAACCTTGTGGATTGCGCCGAGAGT

461 A T A Q L N K E L N N N D L R A M L G D

1381 GCCACAGCACAACTCAACAAGGAGTTGAACAACAACGACTTGAGAGCAATGCTTGGAGAT

481 V M R T I E G P D Y V D Q D E L E R L F

1441 GTGATGCGAACGATTGAAGGACCGGACTATGTGGATCAGGATGAACTTGAAAGACTGTTC

501 N L K L P L N L G D F M P L L H P R K P

1501 AACTTGAAGCTTCCACTGAATCTTGGAGACTTTATGCCACTTCTTCACCCCAGAAAACCA

521 T H R T L I Y R G D L R M Q E G K K G S

1561 ACCCATCGAACACTCATTTATAGGGGAGACCTTAGAATGCAGGAGGGGAAGAAAGGATCA

541 K A D V H C I I F T D M F L I C R K V Q

1621 AAAGCCGATGTGCATTGTATCATCTTCACCGATATGTTTCTGATTTGTCGGAAAGTTCAA

561 G K K D R L K I L K P P I H M G K M M F

1681 GGAAAGAAGGATCGTCTGAAGATTTTGAAGCCTCCAATTCACATGGGAAAGATGATGTTC

581 H Y F A D Q N G F Y L V H L T D F H T A

1741 CACTATTTCGCCGATCAGAATGGATTCTACTTGGTTCATCTCACCGACTTCCACACCGCG

601 Q A L Y S M H T S G P E D T L R W T D M

1801 CAGGCTCTTTACTCGATGCACACGTCGGGGCCAGAAGATACACTTCGATGGACCGACATG

621 L K M A L D E F K K I H R D S W S Q Q N

1861 CTGAAAATGGCACTGGATGAGTTCAAGAAGATCCATAGAGATTCATGGTCACAGCAGAAT

641 Q Q E S P L D E Y G R G F I M E P S S F

1921 CAGCAGGAATCTCCACTCGATGAGTATGGAAGGGGTTTCATTATGGAGCCGAGTAGTTTC

661 Y S Q R M L P P G Y A Q Q Q M P V I H R

1981 TATTCTCAACGAATGTTGCCACCCGGATATGCTCAGCAGCAGATGCCAGTGATTCATAGG

681 K C S S M D S Q A V A A H A H L N Y M H

2041 AAGTGCAGTAGTATGGATTCGCAAGCGGTCGCTGCACATGCTCATTTGAACTACATGCAC

701 R S S A V S S T E Q L D R H S G T D S M

2101 CGAAGCTCTGCAGTCTCCTCCACTGAACAACTCGATCGTCACAGTGGCACGGACAGCATG

721 K C S P P R H K L S V A S C H A N P L S

2161 AAGTGCTCACCACCTCGACATAAACTAAGTGTTGCAAGCTGCCACGCCAACCCCTTATCC

741 S S K S S V D L Y V S L G A E N G D I E

2221 TCTAGTAAAAGCTCAGTGGATCTCTATGTTTCACTTGGAGCTGAAAACGGGGATATCGAG

761 R P R S R S N S S G P E I E G L K Q R S

2281 CGACCACGAAGCAGATCCAACTCTTCTGGTCCTGAAATCGAGGGACTCAAGCAGCGATCG

781 R S S S P D Q K E L G T P A Q I G S P Q

2341 AGAAGCTCTTCTCCAGATCAAAAGGAACTAGGAACCCCAGCACAAATCGGATCTCCACAG

801 T N T P C R D S P T L L I T S D D C V E

2401 ACCAACACTCCGTGTCGAGACTCTCCAACGCTTCTCATCACGTCTGATGATTGTGTTGAG

821 P L Q F G R R F E K R Y H T A D G I D V

2461 CCATTACAATTTGGACGAAGATTCGAGAAACGATATCATACAGCCGATGGAATTGACGTA

841 L K P K I S K L P G A I L K R F S L N G

2521 CTGAAGCCTAAAATATCAAAGCTGCCTGGAGCCATCCTAAAACGATTCTCATTGAACGGA

861 G S G A V G S S C K K L E S S K R N S Q

2581 GGTTCAGGGGCCGTAGGAAGCAGTTGCAAGAAACTGGAGAGCTCGAAAAGGAACTCACAG

881 A S N A A S L D S F G S S T S G I S T A

2641 GCTTCGAATGCTGCTAGTTTGGACTCTTTCGGAAGTTCCACAAGCGGGATTTCCACGGCC

901 S S N N N D P S M E T L T S K L S H I S

2701 TCGTCGAATAACAACGATCCCAGCATGGAAACGTTAACGTCAAAACTCTCCCACATTTCT

921 T I S I N D S P S S I D S Q G T L S I S

2761 ACCATTTCAATCAACGACTCGCCATCTTCCATTGACTCTCAGGGCACGCTGAGCATCAGC

941 L E A P P T V L E K D E D Q H S I E V S

2821 TTGGAAGCTCCACCTACAGTTTTAGAGAAAGATGAAGATCAACACTCGATCGAAGTAAGT

961 V P P P P E L P P P S K T P S P N L P T

2881 GTCCCACCGCCGCCAGAGCTTCCGCCGCCGAGCAAGACCCCGTCACCGAACCTTCCAACG

981 H K P N K S K V H C E E L M K F I Q D N

2941 CATAAACCGAACAAAAGTAAAGTTCATTGTGAGGAACTTATGAAGTTTATTCAGGATAAT

1001 R L E T S D V STOP

3001 CGTCTAGAGACGTCAGACGTCTAA

Figure S1. The *rhgf-2l* cDNA and translated protein sequences. Exons are indicated by alternate yellow and orange highlighting. The nucleotides deleted in *gk216* are indicated in bold. The *rhgf-2* exon sequence identified in this study is boxed. The RhoGEF domain is located between amino acids 280-467 and the PH domain from amino acids 525-628.
