# Supplementary material for: RHGF-2 Is an Essential Rho-1 Specific RhoGEF that binds to the Multi-PDZ Domain Scaffold Protein MPZ-1 in Caenorhabditis elegans
Source: PLoS One. 2012 Feb 20;7(2):e31499. doi: 10.1371/journal.pone.0031499 (PMC3282746; doi:10.1371/journal.pone.0031499)
Supplement: Table S1 — DNA Oligonucleotide Primer List. This table lists the oligonucleotide primers used in the PCR reactions performed in this study. (DOC) [file pone.0031499.s003.doc]

| Gene or Vector | Primer | Sequence (5’ to 3”) | Direction |
| --- | --- | --- | --- |
| T08H4.2 | RS521 | gagcagtagtgttggacatgc | Forward |
| *rhgf-2* | RS523 | aaaaggacaaattcgacaggt | Reverse |
| *rhgf-2* | RS524 | TCTTTGCCGAAGAACAAAGAA | Reverse |
| T08H4.2 | RS796 | TCTCTCTCGACAGTTGACTCCT | Forward |
| T08H4.2 | RS797 | ATCGAATCTAATATGCTCGACGG | Reverse |
| *rhgf-2* | RS945 | ctttctcctgaatgtctctagataattac | Reverse |
| T08H4.2 | RS974 | cctacacaacacagaaattggcac | Forward |
| *rhgf-2* | UT19 | gaaacgccaagaaaaacgag | Reverse |
| *rhgf-2* | UT20 | cacggcacatacaaaaccag | Forward |
| *rhgf-2* | UT25 | tcgaaattcctaattttttgacg | Forward |
| *rhgf-2* | UT28 | CCAAGCGAGGACAATTCTca | Reverse |
| *mpz-1* | UT78 | gccagaggatataatagcagt | Forward |
| *mpz-1* | UT79 | aaaacatggtgcaacagacca | Reverse |
| *mpz-1* | UT80 | ttgtcgtaataagaccggcaa | Forward |
| *mpz-1* | UT81 | TCGACGGTGATAGTGTGTTG | Reverse |
| *mpz-1* | UT82 | ttggtgaaattgggtcgtaaa | Forward |
| *mpz-1* | UT83 | AAAGCAACAGCCTTCTTTCCA | Reverse |
| *mpz-1* | UT84 | ccaactgttttggccgttatt | Forward |
| *mpz-1* | UT85 | ctacccaccccatacacac | Reverse |
| *mpz-1* | UT89 | gccgactccaacatctcaat | Forward |
| *mpz-1* | UT93 | TCTTCGCCCTTAGACACcat**TTGTTGAGGGATACTGTGTGG** | Reverse |
| pJG4-5 | UT131 | GGTTTAATTACCCAAGTTTGAG | Forward |

Table S1. DNA Oligonucleotide Primer List

The nucleotides in bold are complementary to RFP. PCR with this primer generated a product that was used as a template in a subsequent PCR fusion reaction.
